# Phenotype-Driven Next-Generation Sequencing and Structure-Based In Silico Analysis Reveal Disease-Specific Diagnostic Yield and Genotype–Phenotype Correlations in Inherited Kidney Diseases

**DOI:** 10.3390/life16030500

**Published:** 2026-03-18

**Authors:** Savas Baris, Kerem Terali, Serdar Bozlak, Neslihan Yilmaz, Halil Ibrahim Yilmaz, Cuneyd Yavas, Recep Eroz, Mursel Hazaloglu, Kubra Ozen, Alper Gezdirici, Mustafa Dogan, Huseyin Kilic, Senol Demir, Ibrahim Baris

**Affiliations:** 1 Department of Medical Genetics, Aydın City Hospital, Aydin 09020, Turkey; 2 Department of Medical Biochemistry, Faculty of Medicine, Cyprus Health and Social Sciences University, Morphou 99750, Cyprus; 3 Department of Pediatric Genetics, Basaksehir Cam and Sakura City Hospital, Istanbul 34480, Turkey; 4 Department of Pediatric Nephrology, Aydın City Hospital, Aydin 09020, Turkey; 5 Department of Medical Genetics, University of Health Sciences Basaksehir Cam and Sakura City Hospital, Istanbul 34480, Turkey; 6 Department of Molecular Biology and Genetics, Faculty of Engineering and Natural Sciences, Biruni University, Istanbul 34015, Turkey; 7 Department of Medical Genetics, Faculty of Medicine, Aksaray University, Aksaray 68100, Turkey; 8 Division of Genetics, Gelisim Medical Laboratories, Istanbul 34100, Turkey; 9 Department of Pediatric Neurology, Cerrahpasa Faculty of Medicine, Istanbul University, Istanbul 34098, Turkey; 10 Department of Medical Genetics, Van Training and Research Hospital, Van 65000, Turkey; 11 Department of Molecular Biology and Genetics, Koc University, Istanbul 34450, Turkey

**Keywords:** Alport syndrome, polycystic kidney disease, inherited kidney diseases, in Silico analysis, VUS

## Abstract

Background: Inherited kidney diseases represent a genetically and clinically heterogeneous group of disorders affecting both pediatric and adult populations. Advances in next-generation sequencing (NGS) have improved diagnostic precision; however, genotype–phenotype correlations and diagnostic yield vary substantially across disease entities. Methods:We retrospectively evaluated 165 patients referred for genetic testing due to suspected inherited kidney disease. Patients were classified into three clinical groups: polycystic kidney disease, Alport syndrome, and other syndromic patients with inherited kidney diseases. Genetic analysis was performed using NGS with Human Phenotype Ontology–based gene filtering and included evaluation of both single-nucleotide variants and copy number variations. Results: Overall diagnostic yield differed markedly between groups. A molecular diagnosis was achieved in 71.4% of Alport patients, 41.0% of PKD patients, and 70.2% of patients in the Other syndromic group. In the Alport group, variants were identified exclusively in *COL4A3*, *COL4A4*, and *COL4A5*, with pathogenicity and gene involvement correlating with disease severity and the presence of extrarenal manifestations. The PKD group showed predominant involvement of *PKD1*, followed by *PKHD1* and *PKD2*, while a substantial proportion of patients remained genetically negative, reflecting technical and biological complexity. The Other group exhibited pronounced genetic heterogeneity, with variants distributed across multiple genes involved in tubular, glomerular, metabolic, and ciliopathy-related pathways. Computational assessments demonstrated that several variants of uncertain significance (VUS) were located in functionally critical domains and were predicted to disrupt protein stability, intermolecular interactions, or conserved structural motifs, thereby supporting the biological plausibility of their potential pathogenic impact. Conclusions: Phenotype-driven NGS enables effective molecular diagnosis across diverse inherited kidney diseases while revealing disease-specific differences in diagnostic yield and genotype–phenotype correlations. Systematic inclusion of variants of uncertain significance and careful integration of genetic and clinical data are essential for accurate interpretation and long-term patient management. Collectively, this study enhances understanding of inherited kidney diseases and underscores the value of integrating comprehensive genomic and computational approaches into routine nephrogenetic practice.

## 1. Introduction

Inherited kidney diseases (IKDs) constitute a major cause of chronic kidney disease (CKD) across the lifespan and are particularly prominent in pediatric nephrology practice. Large cohort studies have demonstrated that monogenic disorders account for a substantial proportion of childhood-onset CKD and contribute significantly to early adult renal failure [1,2]. Despite advances in clinical diagnostics, the marked genetic and phenotypic heterogeneity of IKDs continues to pose significant diagnostic challenges, especially in patients with early-onset disease or atypical clinical presentations [1,3,4].

The genetic architecture of IKDs is highly complex, involving hundreds of genes that participate in diverse biological pathways, including glomerular basement membrane integrity, podocyte structure, tubular transport, ciliary signaling, and metabolic homeostasis [1,2]. These disorders exhibit all major modes of inheritance—autosomal dominant, autosomal recessive, and X-linked—and pathogenic variants may also arise de novo, frequently contributing to sporadic cases [2,5]. Alport syndrome (AS) and polycystic kidney disease (PKD) illustrate the challenges of genotype–phenotype correlation in nephrogenetics. Alport syndrome, caused by pathogenic variants in *COL4A3*, *COL4A4*, or *COL4A5*, demonstrates wide variability in age at onset, rate of progression, and extrarenal involvement depending on the affected gene, inheritance pattern, and variant type [1,6,7]. Similarly, PKD encompasses a genetically heterogeneous spectrum involving *PKD1*, *PKD2*, *PKHD1*, and additional genes, with disease severity influenced by allelic heterogeneity, modifier effects, and technical challenges in variant detection, particularly for *PKD1* [8,9,10].

The introduction of next-generation sequencing (NGS) has fundamentally transformed the diagnostic evaluation of IKDs. Multiple studies have demonstrated that NGS-based approaches—using targeted gene panels or exome sequencing—significantly increase diagnostic yield compared with traditional stepwise testing strategies [1,2,5]. Nevertheless, diagnostic yield varies considerably across kidney disease categories. Disorders with well-defined genetic etiologies, such as Alport syndrome, generally demonstrate higher mutation detection rates, whereas PKD and clinically heterogeneous renal phenotypes yield lower rates [1,2]. These differences reflect both biological complexity and technical limitations, including incomplete detection of copy number variations (CNVs), deep intronic variants, and low-level mosaicism [2,3].

Phenotype-driven NGS strategies have emerged as an effective means to optimize diagnostic performance. The use of Human Phenotype Ontology (HPO) terms to guide gene selection and variant prioritization has been shown to improve clinical relevance while reducing incidental findings in genetically heterogeneous disorders [2]. A persistent challenge in genomic nephrology is the interpretation of variants of uncertain significance (VUS). Broad next-generation sequencing approaches inevitably identify VUS, particularly in genetically heterogeneous disease groups and in populations underrepresented in reference databases [1,2]. Large nephrogenetic studies have consistently reported a substantial burden of VUS in inherited kidney disease cohorts, highlighting the limitations of sequence-level classification alone and the need for complementary interpretative strategies [1,3]. Importantly, systematic documentation and periodic reinterpretation of VUS are essential, as some variants may later be reclassified as pathogenic or likely pathogenic with accumulating functional, segregation, and population-level evidence [2,3]. In this context, in Silico structural and protein modeling approaches provide a valuable framework to assess the potential functional impact of VUS by evaluating effects on protein structure, stability, and molecular interactions. By integrating in Silico predictions with phenotypic data and inheritance patterns, such approaches enhance biological plausibility, support variant prioritization, and inform future functional and clinical investigations.

In this study, we performed a comprehensive phenotype-driven next-generation sequencing analysis in a cohort of patients with suspected inherited kidney disease. Patients were systematically classified into polycystic kidney disease, Alport syndrome, and a heterogeneous group of other inherited renal disorders. By integrating single nucleotide variant and copy number variation analyses with detailed in Silico structural and protein modeling of selected variants, and by explicitly including pathogenic, likely pathogenic, variants of uncertain significance, and genetically negative cases, we aimed to compare demographic characteristics, diagnostic yield, and genotype–phenotype correlations across disease groups within a clinically meaningful framework relevant to both pediatric and adult nephrology. Collectively, this study provides additional insight into the genetic complexity of inherited kidney diseases and highlights the importance of integrated genomic and computational approaches in evaluation of kidney diseases.

## 2. Materials and Methods

### 2.1. Patients and Data Collection

In the present study, a total of 165 patients were retrospectively evaluated after referral for genetic testing due to suspected inherited kidney disease. Based on clinical phenotype, patients were classified into three groups: polycystic kidney disease (PKD; n = 83), Alport syndrome (n = 35), and other inherited kidney diseases (n = 47). Written informed consent was obtained from all participants or from the parents/legal guardians of pediatric patients. All procedures were conducted in accordance with the principles of the Declaration of Helsinki and were approved by the Aydın Adnan Menderes University Ethics Review Board (Ethical Approval No. 2026-14; Approval Date: 14 January 2026).

A comprehensive clinical history was obtained. Data were collected from all centers (Aydın Maternity and Children’s Hospital (Aydın City Hospital) and Başakşehir Çam and Sakura City Hospital) into a unified, anonymous table. According to the phenotype and the diagnosis, the patients were categorized into three major groups: Alport syndrome, polycystic kidney disease and other syndromic inherited kidney diseases. Patients were categorized according to established clinical diagnostic criteria. Alport syndrome was defined by persistent hematuria with or without proteinuria, family history consistent with collagen IV nephropathy, and/or characteristic extrarenal manifestations such as sensorineural hearing loss or ocular abnormalities, in accordance with previously described diagnostic frameworks [6]. Polycystic kidney disease was defined based on ultrasonographic detection of multiple renal cysts, family history suggestive of autosomal dominant or recessive inheritance, or early-onset cystic kidney disease compatible with PKD-associated genes [10]. Patients who did not fulfill criteria for these two major entities but presented with suspected hereditary renal disorders were categorized into the Other group, which included tubulopathies, ciliopathies, metabolic nephropathies, and hereditary glomerular diseases such as nephrotic syndrome or focal segmental glomerulosclerosis.

Patients underwent genetic evaluation based on a range of clinical features, including: (a) a family history of kidney disease, defined as any family member with urinary abnormalities or impaired kidney function as reported by the parents; (b) abnormalities of kidney function or structure after exclusion of secondary causes; and (c) clinical suspicion of a genetic kidney disease due to early-onset chronic kidney disease in childhood and/or the presence of extrarenal features.

### 2.2. Whole Exome Sequencing

Genomic DNA was extracted from peripheral blood and whole-exome sequencing (WES) was performed using the Twist Human Core Exome kit (Twist Bioscience, South San Francisco, CA 94080, USA). After library enrichment and quality control, the samples were sequenced using the Illumina HiSeq4000 instrument (Illumina Inc., San Diego, CA, USA) with 100 bp paired-end reads at an average sequencing depth of 100×. An average read depth of 20× and 95% coverage, including exon–intron junction boundaries (±10 bp), was evaluated. The raw data analysis was performed as described before [11,12,13]. Raw reads were quality trimmed with Trimmomatic. Surviving high-quality reads were mapped to reference human genome (hg19/GRCh37) using BWA (Burrows-Wheeler Alignment Tool). Picard, a command line java tool to handle SAM and BAM files, was used to mark duplicate reads with appropriate flags. GATK (Genome Analysis Toolkit, 4.6.1.0), a software package to analyze high-throughput sequencing data, was used to call SNPs and indels. Following modules of GATK software package were used in respective order, RealignerTargetCreator, IndelRealigner, BaseRecalibrator, PrintReads, Haplotypecaller, SelectVariants, VariantFiltration, CombineVariants, to call and filter SNPs and indels. The reference human genome (hg19/GRCh37) was used for the analysis. Samples that did not meet the quality thresholds of an average read depth ≥ 20× and 95% coverage were excluded from further analysis. Annotations and ACMG classifications of detected variants were performed using Franklin (https://franklin.genoox.com; accessed on 1 February 2026) and Genomize (https://genomize.com; accessed on 1 February 2026).

Human Phenotype Ontology was used for phenotypic filters and gene sets (Renal insufficiency HP:0000083; Chronic kidney disease HP:0012622; Abnormal renal physiology HP:0012211; Abnormality of the kidney HP:0000077) (HPO, https://hpo.jax.org; accessed on 1 February 2026) was used for gene set filtering. For the PKD and Alport syndrome groups, broad renal phenotype HPO terms were selected to maximize sensitivity during variant prioritization. Specific phenotype terms such as polycystic kidney dysplasia (HP:0000113) and hematuria (HP:0000790) were not applied as exclusive filters in order to avoid excluding genes associated with atypical presentations or overlapping renal phenotypes. Both single-nucleotide variants (SNVs) and copy-number variants (CNVs) were investigated. Coverage-based CNV detection was performed using read-depth analysis integrated within the Genomize analysis platform. High-confidence copy-number variants were interpreted according to ACMG/ClinGen technical standards for constitutional CNV interpretation [14] in conjunction with ACMG sequence variant classification guidelines [15]. Variants with an allele frequency higher than 0.5% were filtered out. Also, variants located outside exon and intron–exon boundary regions (±10 bp), those with sequencing depth below 10×, variants detected only in single-strand reads, variants within homopolymeric regions, and variants with an allelic balance below 20% were filtered out and excluded from further analysis. As integrated in Franklin and Genomize platforms, DANN, MutationTaster, PredictSNP2SIFT, SIFT4G, Provean, Mutation assessor, FATHMM, FATHMM-MKL, FATHMM-XF, MetaSVM, MetaLR, EIGEN, EIGEN PC, LRT, DEOGEN2, MVP, MutPred, REVEL, PrimateAI, and SpliceAI, Human Splicing Finder were used to predict the pathogenicity of variants [12,16].

Confirmation of the identified variant and the segregation within the family was performed using Sanger sequencing on an ABI PRISM 3130 Genetic Analyzer (Applied Biosystems, Thermo Fisher Scientific, Tokyo, Japan). When required, multiplex ligation-dependent probe amplification (MLPA) or array comparative genomic hybridization (array CGH) was performed to confirm CNV alterations.

### 2.3. In Silico Structure-Based Functional Assessment of Selected Variants

Computational analyses were conducted using the solution NMR structure of the third spectrin repeat of human α-actinin 4 (Protein Data Bank ID: 1WLX), the X-ray diffraction structures of the human Fanconi anemia–associated nuclease 1 dimer complexed with DNA (PDB ID: 4REA) [17] and the α4 globular domain of the human type IV collagen homooligomer (PDB ID: 5NB1) [18], the cryo-electron microscopy structure of the human polycystin-1/polycystin-2 complex (PDB ID: 6A70) [19], as well as theoretical models of human chloride channel protein ClC-Kb (AlphaFold Protein Structure Database ID: AF-P51801-F1) and human LIM homeobox transcription factor 1β (AlphaFold ID: AF-O60663-F1) [20]. In addition, a homology model of human paired box protein PAX2 was generated using SWISS-MODEL [21], with the X-ray diffraction structure of the fruit fly PAX2 paired domain in complex with DNA (PDB ID: 1PDN) serving as the structural template. To construct a physiologically relevant homodimeric assembly of human ClC-Kb, the AlphaFold-derived monomeric structure was used to predict quaternary structure using GalaxyGemini [22], guided by the cryo-electron microscopy structure of the bovine ClC-K chloride channel (PDB ID: 5TQQ) as a structural reference. The predicted ClC-Kb homodimer was subsequently subjected to energy minimization to eliminate steric clashes and refine side-chain packing at the dimer interface. The energy-minimized homodimeric ClC-Kb model was then positioned within a flat lipid bilayer using the PPM 2.0 web server [23]. Two chloride ions were incorporated into the final human ClC-Kb homodimer model by structurally aligning it with the bovine ClC-K chloride channel (PDB ID: 5TQQ) and transferring the ion coordinates to the corresponding positions in the human model. To incorporate the missing zinc ion into the relevant zinc-finger module of human LIM homeobox transcription factor 1β, the AlphaFill algorithm was employed [24], followed by energy minimization of the resulting protein–metal complex. In Silico mutagenesis analyses were performed using the Missense3D Portal [25], and favorable noncovalent interactions between residues of interest and neighboring residues were evaluated with Arpeggio [26]. Protein structural elements and residue–residue interaction profiles were visualized using the PyMOL Molecular Graphics System (3.1) (Schrödinger LLC, Portland, OR, USA).

### 2.4. Statistical Analysis

The data were analyzed via the Statistical Package for Social Sciences (IBM Corp., Armonk, NY, USA) 22.0 for Windows 10. The descriptive statistics (number and percentage) were performed. The distribution of data was detected via Shapiro–Wilk test. The Kruskal–Wallis Test was used for pairwise comparisons. Cross-tab analysis was performed to summarize the data in categorical variables and examine the relation between two categorical (nominal or graded) variables. *p* values less than 0.05 were considered statistically significant.

## 3. Results

### 3.1. Comparison of Demographic Parameters Across Disease Groups

We retrospectively evaluated 165 patients referred for genetic testing due to suspected inherited kidney disease. Based on clinical phenotype and diagnosis, patients were categorized into three major groups: Alport syndrome, polycystic kidney disease (PKD), and other syndromic patients with inherited kidney diseases. Patients were comprehensively evaluated with respect to sex distribution, age at disease onset, and genetic diagnostic yield, reflecting potential differences in inheritance patterns, disease mechanisms, and technical challenges associated with genetic testing (Tables S1–S3). No statistically significant differences were observed among the groups in terms of sex distribution (χ^2^ = 0.09; *p* = 0.958). Similarly, no significant differences were found in overall age (χ^2^ = 4.842; *p* = 0.89). When age at disease onset was analyzed, no statistically significant differences were detected among the groups (χ^2^ = 0.616; *p* = 0.735) (Table 1).

### 3.2. Genetic Findings Across Disease Groups

Variants were classified according to ACMG criteria as pathogenic (P), likely pathogenic (LP), or variants of uncertain significance (VUS). Benign and likely benign variants were not reported. Diagnostic yield was defined as the proportion of patients in whom at least one reportable variant was identified, including pathogenic, likely pathogenic, or VUS according to ACMG classification criteria. Molecular diagnosis specifically refers to cases harboring pathogenic or likely pathogenic variants. When family data were available, segregation information was considered during variant interpretation according to ACMG guidelines.

**Figure 1:**
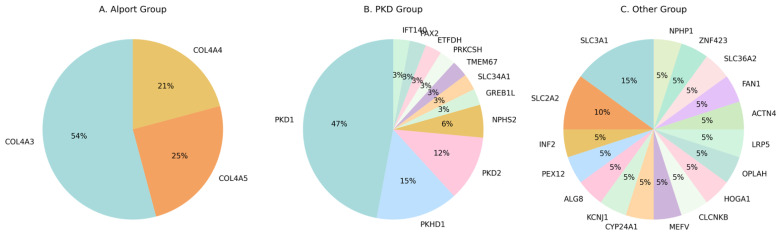
The distribution of detected variants across genes in the study cohort. (**A**) Alport syndrome group showing variants identified in COL4A3, COL4A4, and COL4A5. (**B**) Polycystic kidney disease group showing the predominance of variants in PKD1, followed by PKHD1 and PKD2. (**C**) Other syndromic inherited kidney diseases group demonstrating genetic heterogeneity across multiple genes.

In the Alport group, a total of 35 patients were evaluated using next-generation sequencing. Twenty-five patients (71.4%) harbored at least one reportable variant, while 10 patients (28.6%) were genetically negative under the applied testing strategy. At the variant level, 25 reportable variants were identified, including 9 pathogenic, 10 likely pathogenic, and 6 variants of uncertain significance (VUS). Gene-level analysis demonstrated that variants were restricted to the canonical Alport syndrome genes. The majority of variants were identified in *COL4A3* (n = 13; 54.2%), followed by *COL4A5* (n = 6; 25.0%) and *COL4A4* (n = 5; 20.8%) (Table 1 and Figure 1A). This distribution reflects the known genetic architecture of Alport syndrome, with a predominance of autosomal forms alongside a substantial contribution from X-linked disease.

The PKD group consisted of 83 patients, of whom 34 patients (41.0%) had at least one reportable genetic variant, whereas 49 patients (59.0%) were genetically negative (Table 1 and Figure 1B). In total, 43 reportable variants were identified, including 15 pathogenic, 12 likely pathogenic, and 16 VUS variants. Variants were most frequently detected in *PKD1* (n = 16; 47.1%), followed by *PKHD1* (n = 5; 14.7%) and *PKD2* (n = 4; 11.8%). Additional variants were identified in genes associated with cystic kidney disease or phenotypic overlap, including *NPHS2* (n = 2) and *GREB1L*, *SLC34A1*, *TMEM67*, *PRKCSH*, *ETFDH*, *PAX2*, and *IFT140* (n = 1 each). The predominance of *PKD1* variants is consistent with previous reports and highlights its central role in autosomal dominant polycystic kidney disease.

The Other syndromic patient group included 47 patients with heterogeneous renal phenotypes. A molecular diagnosis was achieved in 33 patients (70.2%), while 14 patients (29.8%) had no reportable variant detected. At the variant level, 34 reportable variants were identified, comprising 8 pathogenic, 12 likely pathogenic, and 14 VUS variants. Gene-level analysis revealed marked genetic heterogeneity. The most frequently affected gene was *SLC3A1* (n = 3; 15.0%), followed by *SLC2A2* (n = 2; 10.0%). Single variants were identified in multiple genes, including *INF2*, *PEX12*, *ALG8*, *KCNJ1*, *CYP24A1*, *MEFV*, *CLCNKB*, *HOGA1*, *OPLAH*, *LRP5*, *ACTN4*, *FAN1*, *SLC36A2*, *ZNF423*, and *NPHP1* (Table S1 and Figure 1C). This wide distribution underscores the extensive genetic diversity of inherited kidney diseases outside classical PKD and Alport categories.

When molecular diagnosis and diagnostic yield were taken into consideration, the PKD group shows a substantially lower diagnostic yield (41%) compared to the Alport (71.4%) and Other (70.2%) groups (*χ*^2^ = 14.829, *p* = 0.001). Across all groups, the diagnostic yield and variant spectrum differed substantially by clinical classification. The Alport and Other groups demonstrated higher mutation-positive rates compared with the PKD group, whereas PKD showed a higher proportion of genetically negative patients. Gene-level analyses revealed disease-specific clustering in PKD and Alport groups, contrasted with pronounced heterogeneity in the Other group. These findings highlight the value of phenotype-driven NGS strategies and comprehensive variant interpretation in diverse nephrogenetic cohorts.

### 3.3. In Silico Structure-Based Functional Assessment of Selected Variants

Variants of uncertain significance (VUS) constituted a notable proportion of the genetic findings in this cohort and were identified across all three disease groups (Tables S1–S3). In the Alport syndrome group, 6 of 25 detected variants (24.0%) were classified as VUS (Table S1), all occurring within the canonical collagen IV genes (*COL4A3*, *COL4A4*, and *COL4A5*). In the PKD group, 6 of 24 variants (25.0%) were classified as VUS (Table S2), primarily involving *PKD1* and other cystic kidney disease–associated genes. The highest proportion of VUS was observed in the heterogeneous Other group, where 13 of 32 variants (40.6%) were classified as VUS (Table S3), reflecting the broad genetic diversity and variable evidence base associated with rare renal disorders.

To further assess the potential functional impact of variants, comprehensive computational analyses incorporating structure-based modeling and targeted in Silico mutagenesis were performed for all missense variants identified across the study cohort, with the exception of those affecting residues located in predicted intrinsically disordered regions. These analyses focused on protein structure, domain architecture, residue conservation, and predicted effects on intramolecular/intermolecular interactions and stability.

**Figure 2:**
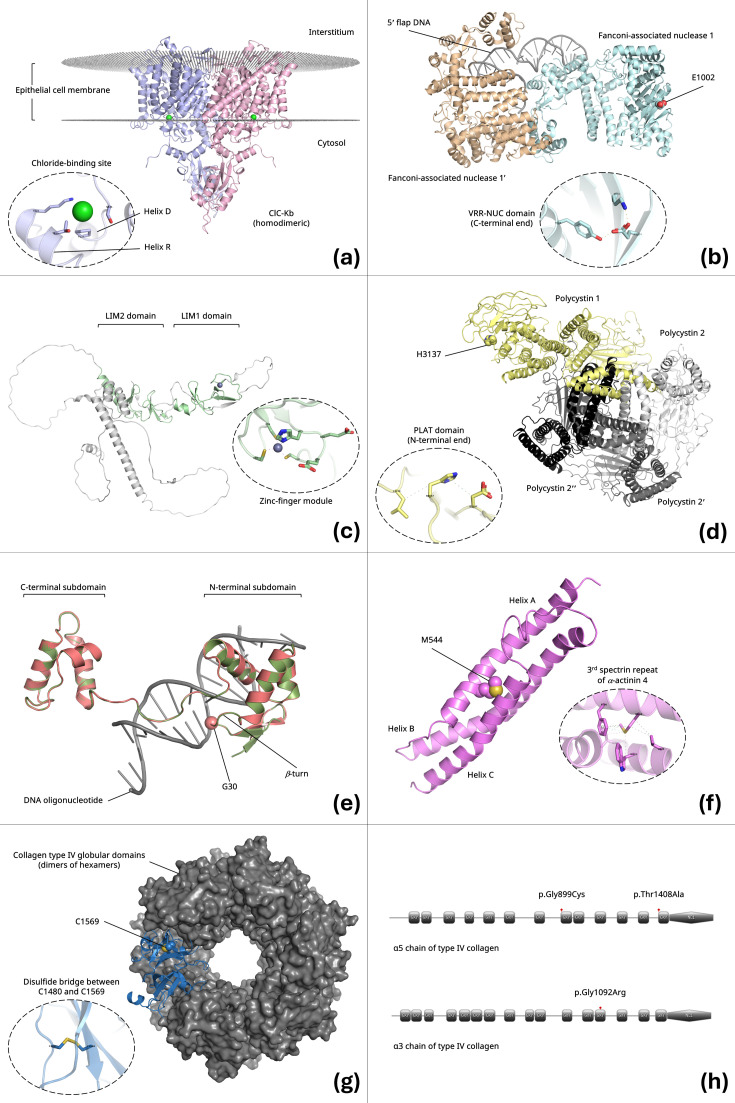
Structural representations of selected proteins implicated in inherited kidney diseases, highlighting the locations and predicted impacts of 10 missense variants classified as VUS or LP in this study. Variant residues are shown as sticks and labeled where appropriate, and predicted noncovalent interactions are indicated by dashed lines. Unless otherwise stated, proteins are displayed as cartoon representations, with interacting residues shown as sticks for clarity. Panels (**a**–**g**) depict three-dimensional structures rendered using PyMOL, based on experimental structures or computational models as described in the Materials and Methods. Panel (**h**) shows a schematic domain representation generated using PROSITE MyDomains. (**a**) Energy-minimized homodimeric model of the human chloride channel ClC-Kb (light blue and light pink cartoon), embedded in a flat lipid bilayer (gray spheres). Each monomer forms an independent chloride-conducting pore. The p.Pro124Leu variant is located on helix D near the chloride-binding site at the cytosolic pore entrance (Cl^−^ shown as a green sphere) and is predicted to cause steric clashes with residues on helix R, potentially affecting pore architecture. (**b**) X-ray structure of the human Fanconi anemia–associated nuclease 1 (FAN1) dimer bound to DNA (PDB ID: 4REA). The p.Glu1002Lys variant lies within the C-terminal region of the VRR-NUC domain (residues 893–1008) and disrupts a hydrogen bond with Tyr822 and a salt bridge with Lys972, potentially compromising core stability. FAN1 subunits are shown as light orange and pale cyan cartoons, and the DNA duplex is shown in gray. (**c**) Model of the human LIM homeobox transcription factor LMX1B showing the N-terminal LIM1 domain (residues 56–111) as a pale green cartoon. The p.Glu79Asp variant affects Glu79 within the His77–Glu78–Glu79–Cys80 loop of the first zinc-finger module (Zn^2+^ shown as a gray sphere coordinated by the Cys3His motif). The substitution shortens the side chain by approximately 1.5 Å and may alter local electrostatic interactions and loop stability. (**d**) Cryo-electron microscopy structure of the human polycystin-1 (PC1)/polycystin-2 (PC2) complex (PDB ID: 6A70). The figure highlights the cytosolic PLAT domain of PC1 (pale yellow cartoon), where the p.His3137Pro variant disrupts a carbon–π interaction with Asp3187 and hydrophobic contacts with Leu3154, potentially destabilizing the local domain fold. (**e**) Homology model of human PAX2 showing the N-terminal subdomain of the paired DNA-binding domain (residues 19–143) bound to DNA (gray cartoon). Human (salmon pink cartoon) and Drosophila (olive green cartoon) proteins are superposed to illustrate conservation of the DNA-binding architecture. The p.Gly30Ser substitution within a β-turn introduces steric hindrance in the DNA minor groove and may impair DNA-binding interactions. (**f**) Solution NMR structure of the third spectrin repeat of human α-actinin-4 (PDB ID: 1WLX), depicted as a three-helix bundle (helices A–C shown as violet cartoons). The p.Met544Ile variant disrupts sulfur–π and carbon–π interactions with Phe540 and hydrophobic contacts with residues on helix C, potentially compromising the stability of the spectrin-repeat bundle. (**g**) X-ray structure of the α4 globular NC1 domain of human type IV collagen (PDB ID: 5NB1). The p.Cys1569Tyr variant abolishes the intramolecular disulfide bond with Cys1480, potentially destabilizing the NC1 domain required for higher-order collagen IV assembly. (**h**) Schematic domain organization of the α5 and α3 chains of type IV collagen. The central collagenous region contains blocks of Gly–X–Y repeats forming the triple helix, followed by the C-terminal NC1 globular domain. Variant positions are indicated: p.Gly899Cys and p.Thr1408Ala in the α5 chain and p.Gly1092Arg in the α3 chain.

In the case of the *CLCNKB* p.Pro124Leu variant, Pro124 is located on helix D of chloride channel protein ClC-Kb (Figure 2a), and our in Silico mutagenesis studies indicated that substitution with leucine cannot be accommodated within the native conformation of the channel without inducing substantial steric clashes with residues on the opposing helix (helix R). Although Pro124 does not appear to participate directly in intracellular Cl^−^ positioning, its replacement by leucine may cause a pronounced reduction in channel function.

Regarding the *LMX1B* p.Glu79Asp variant, Glu79 sits directly within the short His77–Glu78–Glu79–Cys80 loop of the first zinc-finger module of the LIM1 domain (residues 56–111) of LIM homeobox transcription factor 1β (LMX1B) (Figure 2b). Substitution with the shorter aspartate side chain within this backbone-constrained loop is predicted to prevent Asp79 from reaching interacting protein partners. This change may also lead to decreased fold stability due to a significant loss of conformational entropy in the mutated loop.

For the *FAN1* p.Glu1002Lys variant, Glu1002 is located at the C-terminus of the VRR-NUC domain (residues 893–1008) of Fanconi anemia-associated nuclease 1 (FAN1) (Figure 2c). While not an active-site residue, Glu1002 is buried within the protein core. Its substitution with lysine disrupts a critical internal hydrogen bond with Tyr822 and a salt bridge with Lys972, which is predicted to be highly damaging to the structural integrity of the enzyme.

In the *PKD1* p.His3137Pro variant, His3137 is located within the N-terminal half of the cytosolic PLAT domain of polycystin 1 (PC1) (Figure 2d). Upon substitution with proline, local hydrophobic interactions are preserved, but a stabilizing carbon–π interaction with Asp3187 is abolished. The loss of this contact is likely to disrupt the fold and structural integrity of the PLAT domain that participates in high-fidelity protein trafficking and membrane lipid binding.

Concerning the *PAX2* p.Gly30Ser variant, Gly30 is positioned within a *β*-turn of the paired DNA-binding domain (residues 19–143) of the paired box protein PAX2 (Figure 2e). Our analysis indicates that Glu30 is positioned deep within the DNA minor groove. Substitution with the larger serine side chain introduces steric hindrance and is predicted to induce a shift in the peptide backbone, destabilizing the critical *β*-turn-mediated contacts with DNA.

For the *ACTN4* p.Met544Ile variant, Met544 is located on helix A of the third spectrin repeat (residues 519–645) of *α*-actinin 4 (Figure 2f). Substitution with isoleucine results in the loss of a sulfur–π interaction with Phe540 and several hydrophobic contacts with residues Ile619 and Trp623 on helix C. The loss of these stabilizing interactions is likely to compromise the structural integrity of the three-helix bundle.

In the *COL4A4* p.Cys1569Tyr variant, Cys1569 is located within the C-terminal non-collagenous (NC1) globular domain of the α4 chain of type IV collagen (Figure 2g). Substitution of this residue with tyrosine abolishes a conserved intramolecular disulfide bond with Cys1480, which is predicted to destabilize the NC1 globular fold required for proper IV^α345^ hexamer assembly.

The p.Gly899Cys variant in *COL4A5* and the p.Gly1092Arg variant in *COL4A3* both affect strictly conserved glycine residues within the collagenous domains of α5 and α3 chains of type IV collagen, respectively (Figure 2h). These substitutions introduce steric hindrance that obstructs triple-helix formation. Additionally, the mutant Cys899 residue introduces aberrant disulfide-bonding potential. In contrast, the *COL4A5* p.Thr1408Ala variant removes a polar hydroxyl group from the Y position of a Gly–X–Y repeat, which is predicted to cause local destabilization of the triple helix.

## 4. Discussion

In this study, we present a comprehensive genotype–phenotype analysis of a heterogeneous cohort of patients with inherited kidney diseases evaluated using phenotype-driven next-generation sequencing. By stratifying patients into PKD, Alport syndrome, and a genetically heterogeneous Other group, we demonstrate how pathogenicity class, gene involvement, and clinical presentation interact to shape diagnostic yield and disease expression. Our findings are consistent with, and extend, previously published nephrogenetic cohorts that emphasize the utility of broad NGS approaches combined with careful clinical interpretation.

In the Alport group, reportable variants were identified in 71.4% of patients, with variants restricted to the canonical Alport genes *COL4A3*, *COL4A4*, and *COL4A5*, confirming accurate clinical classification and effective phenotype-based gene filtering. The predominance of *COL4A3* variants, followed by *COL4A5* and *COL4A4*, mirrors recent large cohort studies reporting an increasing contribution of autosomal Alport forms alongside classical X-linked disease [1,6]. Patients harboring pathogenic or likely pathogenic variants typically presented with classical renal manifestations, including persistent hematuria, proteinuria, and progressive renal dysfunction. Extrarenal manifestations, particularly sensorineural hearing loss and ocular abnormalities, were more frequently observed in patients with pathogenic variants, especially those involving *COL4A5* or biallelic *COL4A3/COL4A4*, consistent with the systemic distribution of type IV collagen [6].

In contrast, patients with VUS often exhibited milder or incomplete phenotypes, highlighting the importance of cautious interpretation and long-term follow-up for potential variant reclassification. Approximately one-third of Alport patients were genetically negative, a proportion comparable to previously reported cohorts [1]. This likely reflects limitations of current sequencing approaches, including undetected deep intronic variants, regulatory alterations, or complex structural changes, as well as possible phenocopies. Collectively, these observations reinforce the necessity of integrating genetic data with detailed clinical and histopathological findings in the evaluation of suspected Alport syndrome.

The PKD group demonstrated a lower diagnostic yield (41.0%) compared with the Alport and Other groups, with *PKD1* being the most frequently affected gene, followed by *PKHD1* and *PKD2.* This distribution is consistent with prior studies demonstrating that *PKD1* variants are associated with earlier disease onset, more severe cyst burden, and more rapid progression to chronic kidney disease, whereas *PKD2* variants typically confer a milder phenotype with later onset [8,10]. Variants in *PKHD1* were identified in a subset of patients, likely representing autosomal recessive PKD or early-onset cystic disease, which is particularly relevant in pediatric presentations. The identification of additional variants in genes such as *NPHS2*, *GREB1L*, *TMEM67*, *IFT140*, *PRKCSH*, and *PAX2* underscores the phenotypic overlap between PKD and other ciliopathies or congenital renal disorders, a phenomenon increasingly recognized in nephrogenetic studies [10]. A substantial proportion of PKD patients (59.0%) were genetically negative, consistent with published data highlighting ongoing technical challenges in PKD diagnostics—particularly for *PKD1*—due to pseudogene homology, mosaicism, and structural complexity [8]. Polycystic kidney diseases show substantial genetic and phenotypic heterogeneity, and some patients with clinical PKD phenotypes may harbor variants in non-canonical genes or structural variants that are difficult to detect using standard NGS pipelines. These factors likely contribute to the lower mutation detection rate observed in our PKD cohort. These findings emphasize that a negative genetic result does not exclude a clinical diagnosis of PKD and underscore the importance of integrating genetic testing with imaging findings, family history, and longitudinal clinical follow-up.

The Other group encompassed a heterogeneous spectrum of inherited kidney diseases, including tubulopathies, ciliopathies, metabolic nephropathies, and hereditary glomerular disorders. Variant prioritization was performed using phenotype-driven filtering based on broad renal HPO terms in order to maximize sensitivity and avoid exclusion of genes associated with atypical or overlapping renal phenotypes. Although such heterogeneity may influence comparisons of diagnostic yield across disease categories, gene-specific analyses demonstrated that detected variants were consistent with the clinical phenotypes of individual patients, supporting the validity of the phenotype-driven approach. The Other group exhibited the highest genetic heterogeneity and a diagnostic yield of 70.2%, comparable to large multicenter studies of mixed pediatric and adult renal cohorts [1,3]. Variants were distributed across a wide range of genes, including *SLC3A1*, *SLC2A2*, *INF2*, *CLCNKB*, *KCNJ1*, *HOGA1*, *ACTN4*, *FAN1*, *NPHP1*, and others, reflecting diverse pathogenic mechanisms involving tubular transport, glomerular integrity, ciliary function, and metabolic pathways. Patients with pathogenic or likely pathogenic variants generally exhibited clinical features consistent with known gene-associated phenotypes, such as tubular dysfunction, nephrolithiasis, proteinuria, or progressive renal impairment. Genes linked to ciliopathies and structural renal disease were frequently associated with early-onset or syndromic presentations, whereas glomerular genes such as *INF2* and *ACTN4* correlated with proteinuric phenotypes. The relatively high proportion of VUS in this group reflects both the marked genetic diversity and current limitations in variant interpretation, as also reported in comparable published cohorts [1]. Approximately 30% of patients in this group were genetically negative, which may reflect undetected non-coding variants, complex inheritance patterns, or non-monogenic etiologies. Collectively, these findings underscore the importance of ongoing variant reinterpretation and integration of genetic results with detailed clinical phenotyping.

Variants of uncertain significance (VUS) represent a major interpretative challenge in genomic studies of inherited kidney diseases, particularly in cohorts characterized by marked genetic and phenotypic heterogeneity. In the present study, VUS constituted a substantial proportion of detected variants across all disease groups (Tables S1–S3), with the highest burden observed in the heterogeneous Other group. This observation is consistent with previous large nephrogenetic studies and reflects both the expanding application of broad NGS panels and the limited availability of functional and segregation data for many rare renal disease–associated genes [1,3]. To address this limitation, we performed detailed in Silico structural and protein modeling analyses for selected VUS, aiming to provide additional evidence regarding their potential functional relevance.

The integration of in Silico structural modeling with clinical data allowed for a deeper understanding of how subtle amino acid substitutions translate into macroscopic renal dysfunction. Our findings revealed that several VUS-affected residues located in functionally or structurally critical regions of the encoded proteins, including ion channel gating domains, DNA-binding or transcriptional regulatory regions, enzymatically active or stabilizing domains, and collagen triple-helical or NC1 domains. Importantly, many of these variants were predicted to disrupt key molecular interactions, such as hydrogen bonds, salt bridges, disulfide bonds, or conserved glycine residues, suggesting plausible effects on protein stability, folding, or function.

The p.Pro124Leu variant in *CLCNKB* highlights the delicate balance of chloride transport in the distal nephron. ClC-Kb is a passive basolateral channel essential for electrogenic Cl^−^ efflux during transepithelial NaCl reabsorption [27]. Because Pro124 resides on helix D, a structural element linked to pore closure [28], the predicted steric clash with helix R likely locks the channel in a non-functional state. This provides a structural explanation for the experimental observations where similar mutations resulted in residual currents below 50% of wild-type levels [28]. Similarly, the p.Glu1002Lys variant in *FAN1* underscores the importance of DNA repair mechanisms in the kidney. FAN1 is a nuclease involved in resolving DNA interstrand cross-links. Its deficiency in parenchymatous organs like the kidney leads to spontaneous hyperploidy and karyomegalic interstitial nephritis [29]. By disrupting buried hydrogen bonds and salt bridges in the VRR-NUC domain comprising the catalytic PD(X)*_n_*EXK motif [30], the p.Glu1002Lys mutation likely destabilizes the enzyme’s catalytic core, driving the chronic interstitial fibrosis and tubular atrophy characteristic of this condition.

Transcription factors LMX1B and PAX2 are master regulators of kidney development. LMX1B is a podocyte-enriched factor required for the glomerular filtration barrier [31]. While the p.Glu79Asp substitution is conservative, our model suggests that the shortening of the side chain by approximately 1.5 Å prevents the formation of essential salt bridges with partners like LDB1 [32]. This missing link likely reduces the recruitment of the transcriptional machinery necessary for podocyte health. In PAX2, a factor required for the earliest phase of mesenchyme-to-epithelium conversion [33], the p.Gly30Ser variant affects a *β*-turn that fits precisely into the DNA minor groove [34]. The requirement for glycine at this position is strictly conserved across species. For instance, the *undulated* mouse phenotype and similar *Drosophila* mutants demonstrate that replacing this glycine with serine severely impairs DNA-binding capacity [35,36]. Our findings confirm that the larger serine side chain induces a backbone shift, directly interfering with the transcription factor’s ability to regulate kidney cell differentiation.

Several variants targeted the mechanical and structural components of the glomerulus. *α*-actinin 4 (encoded by *ACTN4*) functions as a homodimer that cross-links filamentous actin in podocyte foot processes, allowing them to withstand high capillary pressures [37,38]. The p.Met544Ile variant compromises the three-helix bundle of the spectrin repeat by abolishing sulfur–π and hydrophobic interactions. Such local destabilization may lead to the detachment of podocytes and subsequent focal segmental glomerulosclerosis. Finally, the variants in type IV collagen (*COL4A3*, *COL4A4*, and *COL4A5*) disrupt the defining structural network of the mature glomerular basement membrane (GBM). The IV^α345^ hexamer assembly is governed by the NC1 domain; thus, the loss of the Cys1569–Cys1480 disulfide bond in the *COL4A4* p.Cys1569Tyr variant likely prevents proper network formation [39,40]. Within the collagenous domain, the p.Gly899Cys (*COL4A5*) and p.Gly1092Arg (*COL4A3*) variants disrupt the obligatory glycine register. Because glycine is the only residue small enough to fit within the center of the triple helix, these substitutions sterically hinder triple-helix folding, leading to the thinning and splitting of the GBM observed in Alport syndrome and related nephropathies.

Despite these supportive in Silico findings, structural modeling alone is insufficient to reclassify VUS as pathogenic or likely pathogenic under current ACMG guidelines. In Silico predictions provide supporting evidence (ACMG criterion PP3) but must be interpreted cautiously and in conjunction with clinical phenotype, inheritance pattern, segregation data, population frequency, and, where available, functional assays. Nevertheless, the concordance between predicted structural disruption and patient phenotype in several cases strengthens the biological plausibility of these variants and highlights candidates for future functional validation. The limitation of the present study relates to the use of AlphaFold-derived structural models for proteins lacking experimentally determined structures. Although AlphaFold has demonstrated remarkable accuracy in predicting protein folds, particularly at the level of overall domain architecture, predicted models may be less reliable in flexible regions, inter-domain orientations, and oligomeric assemblies. Furthermore, AlphaFold predictions generally represent a single static conformation and do not fully capture protein dynamics, ligand-induced conformational changes, or interactions with binding partners and membranes. Consequently, while the AlphaFold-based models used here provide valuable structural frameworks for interpreting the potential effects of missense variants, the predicted structural consequences should be interpreted with caution and ideally validated through experimental structural or functional studies.

This study has several limitations that should be considered when interpreting the findings. First, although next-generation sequencing enabled comprehensive analysis of coding variants and copy number variations, certain classes of genomic alterations—such as deep intronic variants, complex structural rearrangements, and low-level mosaic mutations—may not have been fully detected using short-read sequencing approaches. This limitation may partially explain the proportion of genetically unresolved cases, particularly within the PKD group. Second, the Other disease category included a heterogeneous set of renal phenotypes, which reflects real-world clinical nephrogenetic practice but may limit detailed subgroup comparisons. Third, segregation analyses and functional validation studies were not available for all variants, particularly those classified as variants of uncertain significance. While structure-based in Silico modeling provided supportive evidence regarding potential functional impact, such computational predictions cannot replace experimental validation. Finally, this was a single-cohort study, and larger multicenter datasets will be important to further refine genotype–phenotype correlations and improve interpretation of rare variants in inherited kidney diseases. Despite these limitations, this study also has several strengths. It provides a comprehensive genetic analysis of a clinically well-characterized cohort of patients with inherited kidney diseases, integrating next-generation sequencing with detailed phenotypic evaluation. The study also highlights the diagnostic utility of genetic testing across different renal disease categories and demonstrates the value of combining variant interpretation with structural modeling to support the assessment of novel variants. In addition, the inclusion of multiple disease groups allows for comparative insights into the genetic landscape of inherited kidney disorders within a single cohort, contributing valuable data to the growing field of nephrogenetics.

An important aspect of the present study is that the reported diagnostic yield includes not only pathogenic and likely pathogenic variants but also variants of uncertain significance. The inclusion of VUS reflects real-world genomic diagnostics, where variants without definitive classification are frequently encountered, particularly in genetically heterogeneous diseases and populations that are underrepresented in reference databases. However, the contribution of VUS to diagnostic yield should be interpreted cautiously, as their clinical significance remains uncertain and may change with the accumulation of additional evidence. In clinical practice, VUS findings require careful interpretation in the context of detailed phenotypic information, inheritance patterns, and segregation analysis when available. Importantly, as functional studies, population databases, and variant annotation frameworks continue to expand, a proportion of VUS may ultimately be reclassified as pathogenic or benign. Therefore, systematic documentation and periodic re-evaluation of VUS remain essential components of genomic medicine and may further refine the diagnostic interpretation of inherited kidney diseases over time.

Taken together, our findings illustrate the dual role of VUS in inherited kidney disease diagnostics: while they reflect current limitations in variant interpretation, when combined with rigorous in Silico analysis and detailed phenotyping, they offer valuable insights into disease mechanisms and candidate pathogenic variants. Systematic documentation and re-evaluation of VUS, particularly in the context of emerging structural, functional, and clinical data, will be essential to refine genotype–phenotype correlations and improve diagnostic precision in nephrogenetics [1,3].

## 5. Conclusions

In this study, we performed a comprehensive genotype–phenotype analysis of a clinically heterogeneous cohort of patients with suspected inherited kidney diseases using a phenotype-driven next-generation sequencing approach. Stratification into polycystic kidney disease, Alport syndrome, and other inherited kidney disease groups allowed identification of disease-specific genetic architectures, diagnostic yields, and clinical correlations. Diagnostic yield varied substantially by disease category, with lower mutation detection rates in polycystic kidney disease. This lower diagnostic yield likely reflects both biological complexity and technical challenges associated with PKD genetic testing. In particular, the large size and high pseudogene homology of PKD1 complicate variant detection using short-read sequencing technologies. In addition, phenotypic overlap with other cystic kidney disorders and the presence of structural variants or mosaic mutations may further contribute to genetically unresolved cases.

The systematic inclusion of variants of uncertain significance represents a key strength of this study and reflects real-world clinical genetics practice, supporting future reinterpretation as additional evidence emerges. However, the presence of genetically unresolved cases underscores the need for continued methodological refinement and integration of advanced genomic approaches. Overall, our findings support the clinical utility of phenotype-driven next-generation sequencing in inherited kidney diseases and emphasize the importance of integrating genetic, clinical, and longitudinal data to improve diagnosis, counseling, and precision management. Ultimately, the integration of comprehensive genomic data with detailed clinical phenotyping and emerging multi-omics approaches will be essential to advance precision medicine in inherited kidney diseases.

## Figures and Tables

**Table 1:** Demographic characteristics, genetic findings, and family history by disease group.

Disease Group (n)	Female n (%)	Male n (%)	Median Age (Range)	Median Age at Onset (Range)	Number of Reportable Variants			Patients with No Mutation (%)	Patients with Positive Family History (%)
					Pathogenic (%)	Likely Pathogenic (%)	VUS (%)		
Alport Syndrome (35)	19 (54.3%)	16 (45.7%)	14 y (5–57 y)	8 y (6 m–41 y)	9 (36%)	10 (40%)	6 (24%)	10 (28.6%)	14 (56.0%)
PKD (83)	43 (51.8%)	40 (48.2%)	13 y (0–76 y)	8 y (0–53 y)	15 (34.9%)	12 (27.9%)	16 (37.2%)	49 (59.0%)	34 (41.0%)
Other (47)	24 (51.1%)	23 (48.9%)	15 y (1–70 y)	9 y (0–57 y)	8 (23.5%)	12 (35.3%)	14 (41.2%)	14 (29.8%)	29 (42.6%)
Overall (165)	86 (52.1%)	79 (47.9%)	14 y (0–76 y)	8 y (0–57 y)	32 (31.3%)	34 (33.3%)	36 (35.3%)	73 (44.2%)	68 (41.8%)

## Data Availability

The original contributions presented in this study are included in the article Supplementary Materials. Further inquiries can be directed to the corresponding author.

## Supplementary Materials

The following supporting information can be downloaded at: https://www.mdpi.com/article/10.3390/life16030500/s1, Tables S1–S3: Clinic of patients and characteristics of detected variants in patients

## References

[1] Rao J., Liu X., Mao J., Tang X., Shen Q., Li G., Sun L., Bi Y., Wang X., Qian Y. (2019). Genetic spectrum of renal disease for 1001 Chinese children based on a multicenter registration system. Clin. Genet..

[2] Shah M., Shanks M., Packham E., Williams J., Haysmoore J., MacLaren R.E., Németh A.H., Clouston P., Downes S.M. (2020). Next generation sequencing using phenotype-based panels for genetic testing in inherited retinal diseases. Ophthalmic Genet..

[3] Connaughton D.M., Kennedy C., Shril S., Mann N., Murray S.L., Williams P.A., Conlon E., Nakayama M., van der Ven A.T., Ityel H. (2019). Monogenic causes of chronic kidney disease in adults. Kidney Int..

[4] Knoers N., Antignac C., Bergmann C., Dahan K., Giglio S., Heidet L., Lipska-Ziętkiewicz B.S., Noris M., Remuzzi G., Vargas-Poussou R. (2022). Genetic testing in the diagnosis of chronic kidney disease: Recommendations for clinical practice. Nephrol. Dial. Transplant..

[5] Groopman E.E., Marasa M., Cameron-Christie S., Petrovski S., Aggarwal V.S., Milo-Rasouly H., Li Y., Zhang J., Nestor J., Krithivasan P. (2019). Diagnostic utility of exome sequencing for kidney disease. N. Engl. J. Med..

[6] Savige J., Ariani F., Mari F., Bruttini M., Renieri A., Gross O., Deltas C., Flinter F., Ding J., Gale D.P. (2019). Expert consensus guidelines for the genetic diagnosis of Alport syndrome. Pediatr. Nephrol..

[7] Eroz R., Damar I.H., Kılıçaslan O. (2020). Thrombosis risk of Alport syndrome patients: Evaluation of cardiological, clinical, biochemical and genetic factors. Blood Coagul. Fibrinolysis.

[8] Audrézet M.P., Cornec-Le Gall E., Chen J.M., Redon S., Quéré I., Creff J., Bénech C., Maestri S., Le Meur Y., Férec C. (2012). Autosomal dominant polycystic kidney disease: Comprehensive mutation analysis of PKD1 and PKD2 in 700 unrelated patients. Hum. Mutat..

[9] Cornec-Le Gall E., Audrézet M.P., Chen J.M., Hourmant M., Morin M.-P., Perrichot R., Charasse C., Whebe B., Renaudineau E., Jousset P. (2013). Type of PKD1 mutation influences renal outcome in ADPKD. J. Am. Soc. Nephrol..

[10] Bergmann C., Guay-Woodford L.M., Harris P.C., Horie S., Peters D.J.M., Torres V.E. (2018). Polycystic kidney disease. Nat. Rev. Dis. Primers.

[11] Unsel-Bolat G., Bolat H., Citli S., Ozdemir O., Baris I. (2025). Novel RORA variants reveal genotype-phenotype diversity and variable expressivity in neurodevelopmental disorders. Clin. Genet..

[12] Baris S., Ipek R., Baris S.T., Baris I. (2026). Expanding the phenotypic spectrum of NDUFS6-related disease: From neonatal mitochondrial encephalopathy to childhood-onset axonal neuropathy. Int. J. Mol. Sci..

[13] Baris S., Dogan M., Terali K., Gezdirici A., Eroz R., Yucel P.P., Kilic H., Yavas C., Yildirim G., Baris I. (2026). Biallelic truncating DNAH14 variant in siblings with neurodevelopmental disorder and predominant ataxia: Clinical report and literature review. Int. J. Mol. Sci..

[14] Riggs E.R., Andersen E.F., Cherry A.M., Kantarci S., Kearney H., Patel A., Raca G., Ritter D.I., South S.T., Thorland E.C. (2020). Technical standards for the interpretation and reporting of constitutional copy-number variants: A joint consensus recommendation of the American College of Medical Genetics and Genomics (ACMG) and the Clinical Genome Resource (ClinGen). Genet. Med..

[15] Richards S., Aziz N., Bale S., Bick D., Das S., Gastier-Foster J., Grody W.W., Hegde M., Lyon E., Spector E. (2015). Standards and guidelines for the interpretation of sequence variants: A joint consensus recommendation of the American College of Medical Genetics and Genomics and the Association for Molecular Pathology. Genet. Med..

[16] Akkus N., Canbal A., Guneysu S., Gokce E., Duzgun P., Baris İ. (2026). Whole exome sequencing in patients with developmental delay/intellectual disability (DD/ID), epilepsy and the first Turkish patient diagnosed with BCL11A-related intellectual disability. Mol. Genet. Genomic Med..

[17] Zhao Q., Xue X., Longerich S., Sung P., Xiong Y. (2014). Structural insights into 5′ flap DNA unwinding and incision by the human FAN1 dimer. Nat. Commun..

[18] Casino P., Gozalbo-Rovira R., Rodríguez-Díaz J., Banerjee S., Boutaud A., Rubio V., Hudson B.G., Saus J., Cervera J., Marina A. (2018). Structures of collagen IV globular domains: Insight into associated pathologies, folding and network assembly. IUCrJ.

[19] Su Q., Hu F., Ge X., Lei J., Yu S., Wang T., Zhou Q., Mei C., Shi Y. (2018). Structure of the human PKD1-PKD2 complex. Science.

[20] Jumper J., Evans R., Pritzel A., Green T., Figurnov M., Ronneberger O., Tunyasuvunakool K., Bates R., Žídek A., Potapenko A. (2021). Highly accurate protein structure prediction with AlphaFold. Nature.

[21] Waterhouse A., Bertoni M., Bienert S., Studer G., Tauriello G., Gumienny R., Heer F.T., de Beer T.A.P., Rempfer C., Bordoli L. (2018). SWISS-MODEL: Homology modelling of protein structures and complexes. Nucleic Acids Res..

[22] Lee H., Park H., Ko J., Seok C. (2013). GalaxyGemini: A web server for protein homo-oligomer structure prediction. Bioinformatics.

[23] Lomize M.A., Pogozheva I.D., Joo H., Mosberg H.I., Lomize A.L. (2012). OPM database and PPM web server. Nucleic Acids Res..

[24] Hekkelman M.L., de Vries I., Joosten R.P., Perrakis A. (2023). AlphaFill: Enriching AlphaFold models with ligands and cofactors. Nat. Methods.

[25] Ittisoponpisan S., Islam S.A., Khanna T., Alhuzimi E., David A., Sternberg M.J. (2019). Can predicted protein 3D structures provide reliable insights into whether missense variants are disease associated?. J. Mol. Biol..

[26] Jubb H.C., Higueruelo A.P., Ochoa-Montaño B., Pitt W.R., Ascher D.B., Blundell T.L. (2017). Arpeggio: A web server for calculating and visualising interatomic interactions in protein structures. J. Mol. Biol..

[27] Greger R. (2000). Physiology of renal sodium transport. Am. J. Med. Sci..

[28] Louet M., Bitam S., Bakouh N., Bignon Y., Planelles G., Lagorce D., Miteva M.A., Eladari D., Teulon J., Villoutreix B.O. (2017). In Silico model of the human ClC-Kb chloride channel: Pore mapping, biostructural pathology and drug screening. Sci. Rep..

[29] Zhou W., Otto E.A., Cluckey A., Airik R., Hurd T.W., Chaki M., Diaz K., Lach F.P., Bennett G.R., Gee H.Y. (2012). FAN1 mutations cause karyomegalic interstitial nephritis, linking chronic kidney failure to defective DNA damage repair. Nat. Genet..

[30] Deshmukh A.L., Porro A., Mohiuddin M., Lanni S., Panigrahi G.B., Caron M.-C., Masson J.-Y., Sartori A.A., Pearson C.E. (2021). FAN1, a DNA repair nuclease, as a modifier of repeat expansion disorders. J. Huntingt. Dis..

[31] Dreyer S.D., Zhou G., Baldini A., Winterpacht A., Zabel B., Cole W., Johnson R.L., Lee B. (1998). Mutations in LMX1B cause abnormal skeletal patterning and renal dysplasia in nail-patella syndrome. Nat. Genet..

[32] Deane J.E., Mackay J.P., Kwan A.H., Sum E.Y., Visvader J.E., Matthews J.M. (2003). Structural basis for the recognition of LDB1 by the N-terminal LIM domains of LMO2 and LMO4. EMBO J..

[33] Rothenpieler U.W., Dressler G.R. (1993). Pax-2 is required for mesenchyme-to-epithelium conversion during kidney development. Development.

[34] Balling R., Deutsch U., Gruss P. (1988). undulated, a mutation affecting the development of the mouse skeleton, has a point mutation in the paired box of Pax1. Cell.

[35] Chalepakis G., Fritsch R., Fickenscher H., Deutsch U., Goulding M., Gruss P. (1991). The molecular basis of the undulated/Pax-1 mutation. Cell.

[36] Treisman J., Harris E., Desplan C. (1991). The paired box encodes a second DNA-binding domain in the paired homeo domain protein. Genes Dev..

[37] Kaplan J.M., Kim S.H., North K.N., Rennke H., Correia L.A., Tong H.-Q., Mathis B.J., Rodríguez-Pérez J.-C., Allen P.G., Beggs A.H. (2000). Mutations in ACTN4 cause familial focal segmental glomerulosclerosis. Nat. Genet..

[38] Michaud J.L., Lemieux L.I., Dubé M., Vanderhyden B.C., Robertson S.J., Kennedy C.R. (2003). Focal and segmental glomerulosclerosis in mice with podocyte-specific expression of mutant alpha-actinin-4. J. Am. Soc. Nephrol..

[39] Boudko S.P., Bauer R., Chetyrkin S.V., Ivanov S., Smith J., Voziyan P.A., Hudson B.G. (2021). Collagen IV^α345^ dysfunction in glomerular basement membrane diseases. II. Crystal structure of the α345 hexamer. J. Biol. Chem..

[40] Cummings C.F., Pedchenko V., Brown K.L., Colon S., Rafi M., Jones-Paris C., Pokydeshava E., Liu M., Pastor-Pareja J.C., Stothers C. (2016). Extracellular chloride signals collagen IV network assembly during basement membrane formation. J. Cell Biol..

