# Supplementary material for: Phenotype-Driven Next-Generation Sequencing and Structure-Based In Silico Analysis Reveal Disease-Specific Diagnostic Yield and Genotype–Phenotype Correlations in Inherited Kidney Diseases
Source: Life (Basel). 2026 Mar 18;16(3):500. doi: 10.3390/life16030500 (PMC13028123; doi:10.3390/life16030500)
Supplement: Supplementary file 1 [file life-16-00500-s001.zip › TableS1-AlportGroup.pdf]

Table S1. Clinic of patients and characteristics of detected variants in Alport Syndrome

P	Sex	Age	Age at Onset	DC	Referral Complaint	Primary Renal Phenotype	Family History	Additional (Extrarenal findings)	Consanguinity	Gene	Variant cDNA (protein)	Variant Type	Zygosity	IP	ACMG Classification	Novelty
P2	M	56	41	AS	PKD	Hypertension	PKD on mother and two siblings	Sensorineural hearing loss	NA	COL4A4	c.1321_1369+3del (p?)	Splice	HT	AD	P	rs1553676221
P35	F	18	8	AS	USG shows abnormal kidney, Proteinuria, Cortical cyst	Renal anomaly	two cousins	-	NA	COL4A3	c.172G>A (p.Gly58Ser)	Missense	HT	AR/ AD	P (PS4,PM2,PM5, PP2,PP3,PP5)	rs184730597
P48	M	6	5	AS	Burning, Pain, Urinary retention	PKD	PKD on sister	-	NA	COL4A3<br>EHHADH	c.172G>A (p.Gly58Ser)<br>c.1984G>A (p.Gly662Arg)	Missense<br>Missense	HT<br>HT	AR/ AD<br>AD	P (PS4,PM2,PM5, PP3,PP2,PP5)<br>VUS (PM2,PP3)	rs184730597<br>rs147199442
P100	F	14	7	AS	Incidental	Proteinuria	Nephrolithiasis in siblings	asthma	Yes	COL4A3	c.3902G>A (p.Gly1301Asp)	missense	HT	AD, AR	LP (PS4, PM2, PP2, PP3	rs1475469085
P112	F	48	38	AS	FSGS	Focal Segmental Glomerulosclerosis	Sister with FSGS, proteinuria in two children	cholelithiasis	-	COL4A3	c.3274G>A (p.Gly1092Arg	Missense	HT	AD, AR	LP (PM5, PM1, PP2, PP3)	Novel
P117	F	11	9	AS	polycystic kidneys	polycystic kidneys	-	VSD	-	COL4A4	c.2320G>C (p.Gly774Arg)<br>c.4394G>A (p.Gly1465Asp)	Missense<br>Missense	Compound HT	AD, AR	P (PM2, PM3, PM5, PP1, PP2, PP3)<br>LP (PM2, PP2, PP3, PP5)	rs569681869<br>rs533297350
P125	M	21	15	AS	Nephrotic syndrome	Nephrotic syndrome, FSGS	Father in same phenotype	hepatosplenomegaly	yes	COL4A5	c.3955C>T (p.Arg1319Trp)	Missense	Hemizygous	XLD	VUS (PM2, PP2)	rs138903372
P130	F	16	10	AS	Anemia, Fatigue	renal failure	-	Sensorineural hearing loss,	second degree	COL4A3	c.4153+1del (p.?)	Truncating	HM	AD, AR	LP (PVS1, PM2)	Novel
P131	M	7	6 m	AS	Detected in postnatal follow-ups	PKD	Multiple renal cyst in mother	-	-	COL4A1	c.1807C>T (p.Pro603Ser)	Missense	HT	AD, AR	LP (PM2, PM1, PP2, PP3)	rs747585517
P138	F	10	2	AS	generalized edema	Nephrotic syndrome	-	-	-	COL4A5	c.4222A>G (p.Thr1408Ala)	Missense	HT	XLD	VUS (PM2, PP2)	Novel
P139	F	23	10	AS	Nephrotic syndrome	Nephrotic syndrome	-	Pancytopenia, Dysmorphism, Bleeding diathesis	-	COL4A3	c.4421T>C (p.Leu1474Pro)	Missense	HT	AD	VUS (PP2, PP3, PP5, BP6, BS1)	rs200302125
P140	F	14	10	AS	generalized edema	Nephrotic syndrome	-	Short Stature, GH deficiency	second degree	COL4A3	c.4153+1del (p.?)	splice junction loss	HM	AD, AR	LP (PVS1, PM2)	Novel
P142	F	5	9m	AS	generalized edema	Nephrotic syndrome	-	Thrombosis, PFO	NA	COL4A3	c.4123C>T (p.Pro1375Ser)	Missense	HT	AD, AR	VUS (PM2, PP2)	Novel
P143	M	11	5	AS	Nephrotic syndrome, FSGS	Nephrotic syndrome, FSGS	-	-	-	COL4A5	c.1061C>T (p.Thr354Ile)	Missense	HT	XLD	VUS (PM2, PP2)	Novel

P145	F	23	7	AS	CKD	CKD	-	Sensorineural hearing loss, moderate myopia, cataract	-	COL4A3	c.40_63del p.(Leu14_Leu21del)	in frame del	HM	AD, AR	P (PS3, PM2, PM3, PM4, PP1)	rs876657397
P146	M	57	37	AS	Microscopic hematuria, proteinuria	AS	CKD in father and uncle	High myopia	-	COL4A4	c.4706G>A (p.Cys1569Tyr)	Missense	HT	AD, AR	VUS (PM2, PP2, PP3)	Novel
P147	F	37	27	AS	proteinuria	proteinuria	NA	NA	-	COL4A3	c.3321_3329del (p.Ser1108_Gly1110del)	Noncoding	HT	AD, AR	P (PS4, PM2, PM4, PM1)	rs756539994
P148	M	32	8	AS	hematuria, proteinuria	hematuria, proteinuria	Nephrolithiasis in her mother and siblings. CKD in maternal grandmother	-	Same region	COL4A3	c.1006G>T (p.Gly336Cys)	Missense	HT	AD, AR	P (PS4, PM1, PM2	rs1559873550
P149	F	36	20	AS	hematuria	hematuria	Same phenotype in her brother	NA	NA	COL4A3	c.4347_4353del (p.Arg1450ValfsTer77)	frameshift	HT	AD, AR	P (PS4, PVS1, PM2	rs748026887
P150	M	38	20	AS	hematuria	hematuria	Same phenotype in her brother	NA	NA	COL4A3	c.4347_4353del (p.Arg1450ValfsTer77)	frameshift	HT	AD, AR	P (PS4, PVS1, PM2	rs748026887
P151	F	24	17	AS	proteinuria	AS	Same phenotype in three siblings	-	-	COL4A5	c.2695G>T (p.Gly899Cys)	Missense	HT	XLD	LP (PM1, PM2, PM5, PP2, PP3)	Novel
P152	F	5	2	AS	Macroscopic hematuria	Macroscopic hematuria	NA	NA	NA	COL4A5	c.1817G>A (p.Gly606Glu)	Missense	HT	XLD	LP (PM1,PM2, PM5, PP2, PP3, PP5)	rs2147813326
P153	F	9	4	AS	hematuria, proteinuria	AS	End stage renal failure in brother	myopia	third degree	COL4A4	c.81_86del (p.Ile29_Leu30del)	in frame del	HT	AD, AR	LP (PM2, PM3, PM4)	rs771943519
P154	F	37	28	AS	segregation	segregation	Alport syndrome in her child(f)	NA	NA	COL4A5	c.1817G>A (p.Gly606Glu)	Missense	HT	XLD	LP (PM1, PM2, PM5, PP2, PP3, PP5)	rs2147813326
P155	M	9	5	AS	Microscopic hematuria	Microscopic hematuria	-	Growth restriction	third degree	COL4A4	c.2870G>A (p.Gly957Glu)	Missense	HT	AD, AR	LP (PM2, PM5, PP3, PP5)	rs1559503562
P156	M	5	3	AS	Microscopic hematuria	Microscopic hematuria	-	-	-	negative AS pane	-	-	-	-	-	-
P157	M	14	8	AS	Microscopic hematuria	Microscopic hematuria	NA	NA	NA	negative AS pane	-	-	-	-	-	-
P158	F	7	5	AS	Microscopic hematuria	Microscopic hematuria	-	-	-	negative AS pane	-	-	-	-	-	-
P159	M	12	9	AS	hematuria	hematuria	-	-	-	negative AS pane	-	-	-	-	-	-
P160	M	1	0,6	AS	proteinuria	proteinuria	-	Anterior lenticonus, Sensorineural	Same region	negative AS pane and CES result	-	-	-	-	-	-

										possible mitochondrial disease (Pierson)						
P161	F	10	5	AS	proteinuria	proteinuria	NA	NA	NA	negative AS pane	-	-	-	-	-	-
P162	M	11	6	AS	hematuria	hematuria	NA	NA	NA	negative AS pane	-	-	-	-	-	-
P163	M	37	36	AS	hematuria, proteinuria	hematuria, proteinuria	-	-	Same region	negative AS pane	-	-	-	-	-	-
P164	F	33	30	AS	Microscopic hematuria	Microscopic hematuria	-	Microadenoma	-	negative AS pane	-	-	-	-	-	-
P165	M	47	44	AS	hematuria, proteinuria	hematuria, proteinuria, CKD	-	ischemic stroke	-	negative AS pane	-	-	-	-	-	-

M:Male, F:Female, P: Patient, DC: Disease Classification, AS: Alport Syndrome, PKD: Polycystic Kidney Disease, CKD: Chronic Kidney Disease, HM: Homozygous, HT: Heterozygous, AD: Autosomal Dominant, AR: Autosomal Recessive
