# Supplementary material for: Phenotype-Driven Next-Generation Sequencing and Structure-Based In Silico Analysis Reveal Disease-Specific Diagnostic Yield and Genotype–Phenotype Correlations in Inherited Kidney Diseases
Source: Life (Basel). 2026 Mar 18;16(3):500. doi: 10.3390/life16030500 (PMC13028123; doi:10.3390/life16030500)
Supplement: Supplementary file 1 [file life-16-00500-s001.zip › TableS2-PKDGroup.pdf]

Table S2. Clinic of patients and characteristics of detected variants in PKD groups

P	Sex	Age	Age at Onset	DC	Referral Complaint	Primary Renal Phenotype	Family History	Additional (Extrarenal findings)	Consang uinity	Gene	Variant cDNA (Protein)	Variant Type	Zygosity	IP	ACMG Classification	Novelty
P1	M	71	53	PKD	PKD	CRF	NA		NA	PKHD1	c.6335A>G (p.Tyr2112Cys)<br>c.415G>A (p.Val139Ile)	Missense	Comp HT	AR	VUS (PM2,PP3)<br>VUS (PM2,BP4)	rs1197586759<br>rs747975201
P3	M	16	16	PKD	PKD	Pyelonephritis	PKD on Mother	-	NA	PKD1	c.7299_7318dup (p.Asp2440Glyfs*187)	Nonsense	HT	AD	LP (PVS1,PM2)	Novel
P4	M	2	2	PKD	Cystic kidneys, bleeding diathesis	Cystic Erosion on USG	PKD on one sibling	-	NA	GREB1L<br>PKD1	c.347C>T (p.Thr116Ile)<br>c.5645C>T (p.Thr1882Met)	Missense<br>Missense	HT<br>HT	AD<br>AD	LP (PM2,PP2,PP5)<br>VUS (PM2)	rs1333304296<br>rs772716160
P5	F	2	1,5	PKD	PKD	Cystic Erosion on USG	-	-	NA	SLC34A1<br>PKD1	c.1006+1G>A (p.?)<br>c.7852G>A (p.Val2618Met)	Splice<br>Missense	HT<br>HT	AR/ AD<br>AD	P (PS4,PVS1,PM2)<br>VUS (PM2)	rs200095793<br>rs376969316
P6	F	10	8	PKD	Abdominal pain, Cyst	PKD	PKD on mother	-	NA	PKD2	c.916C>T (p.Arg306*)	Nonsense	HT	AD	P (PS4,PVS1,PM2)	rs200001068
P8	M	30	18	PKD	Painful urination	PKD	NA	NA	NA	PKD1	c.3877G>A (p.Val1293Ile)	Missense	HT	AD	VUS (PM2)	rs774353552
P9	M	10	9	PKD	PKD	PKD	-	-	NA	PKD1	c.9410A>C (p.His3137Pro)	Missense	HT	AD	LP (PM2,PM5,PM1)	Novel
P10	M	18	16	PKD	PKD	PKD	-	-	NA	PKHD1	c.10756_10759delAACT (p.Asn3586Serfs22)	Frameshift	HT-Carrier	AR	LP (PVs1,PM2,PP5)	rs1195276132
P11	F	11	11	PKD	PKD	PKD	PKD on one sibling	-	NA	PKD2	c.1081C>T (p.Arg361Ter)	Nonsense	HT	AD	P (PS4,PVS1,PM2)	rs1578130676
P13	F	11	NA	PKD		-	CRF on father	-	NA	PKD1	c.6493C>T (p.Gln2165*)	Nonsense	HT	AD	P (PS4,PVS1,PM2)	rs1485307916
P16	F	22	NA	PKD	Renal Colic	PKD on USG	Isolated renal cyst history on mother	-	NA	PKD1	c.359+4dup (p.?)	splice	HT	AD	VUS (PM, PP3)	Novel
P17	M	Pre nata l	NA	PKD	Abnormal USG, Suspected Ciliopathy	PKD on USG	-	-	NA	TMEM67	c.1140del (p.Pro381LeufsTer6)	Frameshift	HOM	AR	LP (PVS1, PM2)	Novel

P19	M	69	41	PKD	Renal Failure	PKD	NA	hyperoxaluri a, hypertension	NA							
P20	F	53	52	PKD	Renal Failure	PKD	NA	headache	NA							
P21	M	5	1,5	PKD	PKD	PKD	PKD on father	-	NA							
P25	F	11	7	PKD	PKD	PKD	PKD on mother	-	NA							
P26	M	5	3	PKD	PKD	PKD	-	-	NA	PKD1	c.7633C>T (p.Pro2545Ser)	Missense	HT	AD	VUS (PM2,BP4)	rs1328959876
P27	F	9	9	PKD	Frequent Urinary Tract Infections	PKD	-	-	NA							
P28	F	16	15	PKD	PKD	PKD	PKD on brother	-	NA							
P29	F	8	7	PKD	Polycystic kidney	PKD	-	-	NA							
P30	F	12	12	PKD	PKD	PKD	PKD on mother	-	NA							
P31	M	13	9	PKD	Back pain, Frequent urinary tract infection	PKD on USG	PKD on sibling	-	NA							
P32	F	14	4	PKD	PKD	PKD	-	-	NA							
P33	F	18	18	PKD	PKD	PKD	-	-	NA							
P36	M	2	2	PKD	PKD	PKD	PKD one sibling	-	NA	PKHD1	c.525del (p.Asp175Glufs*4) c.5275G>C (p.Gly1759Arg)	Nonsense Missense	Comp HT	AR	P (PVS1,PM2,PP5) VUS (PM2,PP3)	rs1810924520 rs398124488
P38	F	14	NA	PKD	Stone in urinary system, Infection	PKD on USG	-	-	NA							
P39	M	17	16	PKD	PKD	PKD	-	-	NA							
P40	M	7	7	PKD	Frequent urinary tract infection	PKD	PKD on mother, father, sibling and aunt	nausea and vomiting	+	PKD1 PKD2	c.7622dup (p.Arg2544Glnfs*51) c.2189G>A (p.Arg730Gln)	Nonsense Missense	HT HT	AD AD	P (PVS1,PM2,PP5) VUS (PM2,BP4)	Novel rs768360131

P41	F	2	6m	PKD	PKD	PKD	PKD on mother, father and aunt	-	+	PKD1<br>PKD2	c.7622dup (p.Arg2544Glnfs*51)<br>c.2189G>A (p.Arg730Gln)	Nonsense<br>Missense	HT<br>HT	AD<br>AD	P (PVS1,PM2,PP5)<br>VUS (PM2,BP4)	Novel<br>rs768360131
P43	F	17	12	PKD	Back pain, Frequent urinary tract infection	PKD	PKD on mother and uncle with crf	-	NA							
P44	F	16	15	PKD	Painful urination	PKD	-	joint pain, weakness	NA							
P45	M	2	2	PKD	PKD	PKD on USG	-	-	NA							
P46	M	3	1	PKD	Frequent urinary tract infection	PKD	-	-	NA							
P47	M	10	7	PKD	Painful urination	PKD	-	-	NA							
P49	M	13	7	PKD	Abdominal pain, fever, Urinary tract infection	PKD	-	-	NA							
P50	F	1	1	PKD	PKD	PKD	PKD on sibling	-	NA							
P52	F	13	12	PKD	Burning, Pain, Urinary retention	PKD	-	-	NA							
P53	F	17	14	PKD	PKD	PKD	-	-	NA							
P54	M	7	5	PKD	Back pain, Frequent urinary tract infection	PKD	-	-	NA							
P55	F	1	6m	PKD	PKD	PKD	-	-	NA							
P56	F	16	16	PKD	PKD	PKD	-	-	NA							
P58	M	11	9	PKD	PKD	PKD	-	-	NA	PKD1	c.4786_4787insAG (p.Thr1596Lysfs*38)	Nonsense	HT	AD	LP (PVS1,PM2)	Novel
P59	F	20	11	PKD	PKD	PKD	-	-	NA	PKD1	c.12310_12313del(p.Val4104Phefs*93)<br>c.12607C>T (p.Arg4203Trp)	Nonsense<br>Missense	HT<br>HT	AD<br>AD	LP (PVS1,PM2)<br>VUS (PM2)	Novel<br>rs771255288

P60	F	9	8	PKD	PKD	PKD	PKD on mother and sister	-	NA							
P61	M	2	2	PKD	Urinary disorder	-	-	-	NA	PKD1	c.3667G>A (p.Val1223Met)	Missense	HT	AD	VUS (PM2,PP3)	rs749092859
P62	F	18	16	PKD	PKD	PKD	PKD on father	-	NA							
P63	F	4	2,5	PKD	PKD	PKD	-	-	NA							
P65	F	2	8m	PKD	PKD	PKD	-	-	NA							
P66	F	17	9	PKD	PKD	PKD	PKD on sister	-	NA	PRKCSH	c.374_375del (p.Glu125Valfs*21)	Nonsense	HT	AD	P (PS4,PVS1,PM2,PP5)	rs779685748
P67	M	15	11	PKD	PKD on USG	PKD	PKD on father	-	NA							
P68	M	63	48	PKD	PKD on USG	PKD	NA	hypertension, proteinuria	NA	ETFDH	c.679C>A (p.Pro227Thr)	Missense	HT	AR	P (PM3,PM2,PM5,PP3,PP2,PP5)	rs141407224
P69	F	31	31	PKD	PKD	PKD	NA	NA	NA							
P70	F	42	33	PKD	PKD on USG	PKD	PKD on brother, two sister and grandfather	NA	NA							
P71	M	9	5	PKD	PKD	PKD	-	-	NA							
P74	F	1	5m	PKD	PKD	PKD	-	-	NA							
P75	M	3	3	PKD	PKD	PKD	-	-	NA	NPHS2	c.353C>T (p.Pro118Leu)	HT-Carrier		AR	P (PM3,PM1,PP2,PM2,PP3,PP5)	rs869025495
P76	M	15	12	PKD	Proteinuria	PKD	-	-	NA							
P78	F	22	15	PKD	PKD	PKD	-	-	NA							
P79	F	14	14	PKD	PKD	PKD	-	-	NA							
P80	M	18	11	PKD	Proteinuria, Bleeding	PKD	-	-	NA							

P81	M	3	2	PKD	Bloody urine, Pain, Fever	PKD	PKD on father, uncle and grandfather	-	NA	PAX2<br>PKD1	c.88G>A (p.Gly30Ser)<br>c.11017-10C>A (p.?)	Missense<br>Splice	HT<br>HT	AD<br>AD	LP (PM1,PP2,PM2,PP3)<br>P (PS4,PP1,PS3,PM2,PP3,PP5)	Novel<br>rs555703777
P82	F	16	10	PKD	Proteinuria	PKD	-	-	NA	NPHS2	c.686G>A (p.Arg229Gln)	Missense	HOM	AR	VUS (PM5,PP2,BA1,BS2,PP5)	rs61747728
P83	M	24	21	PKD	PKD	PKD	NA	NA	NA							
P84	M	11	7	PKD	Right side pain	PKD	Brother and grandfather with kidney failure	-	NA							
P86	M	67	47	PKD	PKD	PKD	NA	NA	NA	IFT140	c.1041_1042del (p.Val348Serfs*135)	Nonsense	HT	AD	P (PM3,PVS1,PM2,PP5)	Novel
P88	M	5	3	PKD	PKD	PKD	-	-	NA							
P89	M	10	5	PKD	Burning sensation when urinating	PKD	-	-	NA							
P90	M	16	8	PKD	PKD	PKD	PKD on father	-	NA	PKD1	c.2085dup (p.Ala696Argfs*18)	Nonsense	HT	AD	P (PS4,PVS1,PM2,PP5)	rs1567212531
P91	M	76	53	PKD	Recurrent renal stone	Renal Colic	PKD on father and uncle	Vitamin D deficiency	NA	PKHD1	c.10666C>T (p.Arg3556Cys)<br>c.4870C>T (p.Arg1624Trp)	Missense<br>Missense	Comp HT	AR	VUS (PM2)<br>LP (PM3,PM2,PP5)	rs201534300<br>rs200391019
P93	F	4	4	PKD	PKD	PKD	PKD on brother	-	NA							
P94	M	4	2	PKD	Urinary tract infection	PKD on USG	-	-	NA							
P95	F	7	3	PKD	Urinary tract infection	PKD on USG	-	-	NA							

P96	F	7	5	PKD	PKD	PKD on USG	PKD on mother and sibling	-	NA							
P97	F	0	newborn	PKD	PKD	PKD on USG	-	-	NA							
P98	M	13	7	PKD	Recurrent renal stone	PKD on USG	PKD on mother and uncle	Vitamin D deficiency	NA							
P99	F	41	38	PKD	CRF	PKD on USG	NA	NA	NA							
P101	F	2	6m	PKD	Incidental	Cystic kidneys	Bilateral hydronephrosis in sister	-	Yes	PKD1	c.3122C>T (p.Ala1041Val)	missense	HT	AD	VUS (PP3, PM2)	rs777881528
P110	F	22	15	PKD	Cystic kidneys, bleeding diathesis	Cystic kidneys, bleeding diathesis	CKD in paternal grandmother	Hepatic cysts, bleeding diathesis	-	PKHD1	c.5513A>G (p.Tyr1838Cys)<br>c.4870C>T (p.Arg1624Trp)	missense	Compound HT	AR	P (PM2, PM3, PP3)<br>LP (PM3, PP2)	rs777999875<br>rs200391019
P115	F	22	21	PKD	Detected during pregnancy follow-ups	polycystic kidneys	Isolated renal cyst in mother	-	-	PKD1	c.359+4dup (p.?)	SPLİCE-SİTE	HT	AD	VUS (PM2, PP3)	Novel
P126	M	8	6	PKD	PKD	PKD	multiple renal cyst in mother	-	-	PKD1	c.10993A>T (p.Lys3665Ter)	Nonsense	HT	AD	LP (PVS1,PM2)	Novel
P127	F	60	30	PKD	PKD	PKD	PKD on father, sister and father's side also had the same phenotype	arrhythmia	-	PKD2	c.1095-2A>G (p.?)	Spilce Site	HT	AD	LP (PVS1,PM2)	Novel, PMID: 32477541
P128	M	42	35	PKD	PKD	PKD	PKD in mother and brother	-	-	PKD2	c.1837C>T (p.Gln613Ter)	Nonsense	HT	AD	P (PS4, PVS1, PM2)	rs2110127334
P129	F	15	8	PKD	PKD	PKD	NA	DM	-	PKD1	c.856_862del (p.Gly287*)	Nonsense	HT	AD	P (PS4, PVS1, PM2)	rs1555459108

M: Male, F: Female, P: Patient, DC: Disease Classification, AS: Alport Syndrome, PKD: Polycystic Kidney Disease, CKD: Chronic Kidney Disease, HM: Homozygous, HT: Heterozygous, AD: Autosomal Dominant, AR: Autosomal Recessive
