# Supplementary material for: Phenotype-Driven Next-Generation Sequencing and Structure-Based In Silico Analysis Reveal Disease-Specific Diagnostic Yield and Genotype–Phenotype Correlations in Inherited Kidney Diseases
Source: Life (Basel). 2026 Mar 18;16(3):500. doi: 10.3390/life16030500 (PMC13028123; doi:10.3390/life16030500)
Supplement: Supplementary file 1 [file life-16-00500-s001.zip › TableS3-OtherGroup.pdf]

Table S3. Clinic of patients and characteristics of detected variants in Other groups

| P | Sex | Age | Age at Onset | DC | Referral Complaint | Primary Renal Phenotype | Family History | Additional (Extrarenal findings) | Consan guinity | Gene | Variant cDNA (Protein) | Variant Type | Zygosit y | IP | ACMG Classification | Novelty |
|---|---|---|---|---|---|---|---|---|---|---|---|---|---|---|---|---|
| P7 | M | 57 | 57 | O | CRF | CRF | NA | proteinuria | NA | PKD2 | c.2019+5G>A | Splice | HT | AD | LP (PS4,PM2,PP3) | rs1578142944 |
| P12 | M | 11 | NA | O | Isolated proteinuria | - | - | - | NA | CUBN | c.8465C>T (p.Pro2822Leu) | Missense | HOM | AR | VUS (PM2) | rs776663892 |
| P14 | M | 17 | NA | O | Cystine stones in urine | - | - | - | NA | SLC3A1 | c.1973dup (p.Gln659Profs*7) | Nonsense | HOM | AR | LP (PVS1,PM2) | rs2466023457 |
| P15 | M | 10 | NA | O | Lethargy, Fatigue | - | - | - | NA | ATP6V1B1 | c.118+5G>T | | HOM | AR | VUS (PM2,PP3) | Novel |
| P18 | M | | NA | O | | - | - | - | NA | | | | | | | |
| P22 | M | 40 | 27 | O | Headache, Weight loss | distal renal tubular acidosis | - | Dysmorphism, Bleeding diathesis | + | | | | | | | |
| P23 | F | 47 | 41 | O | Renal tubular acidosis | distal renal tubular acidosis | NA | hypertension | NA | | | | | | | |
| P24 | F | 69 | 57 | O | Renal tubular acidosis | Acidosis, Respiratory difficulty | NA | DM, arrythmia | NA | | | | | | | |
| P34 | M | 18 | 11 | O | Protein loss | Nephrotic syndrome | - | - | NA | ZNF423 | c.2762C>T (p.Pro921Leu) | Missense | HT | AR/ AD | VUS (PM2) | rs200585917 |
| P37 | F | 2 | 1 | O | PKD | One kidney | - | - | NA | SLC36A2 | c.1229T>G (p.Val410Gly) | Missense | HT | AD | VUS (PM2,PP3) | rs200334307 |
| P42 | F | 8 | 5 | O | Multi cystic dysplastic kidney | Multi cystic dysplastic kidney | - | - | NA | | | | | | | |
| P51 | M | 70 | 41 | O | CFR | KBY | PKD on mother, sibling, uncle | nausea and vomiting | NA | | | | | | | |
| P57 | F | 10 | 8 | O | Multic ystic dysplastic kidney | Multi cystic dysplastic kidney | - | - | NA | | | | | | | |
| P64 | F | 15 | 15 | O | Proteinuria | Renal Failure | - | Renal pain | NA | | | | | | | |
| P72 | F | 11 | 10 | O | Back pain, Renal pain | - | PKD on mother | - | NA | | | | | | | |
| P73 | F | 31 | 26 | O | Proteinuria, Stone | Nephrotic syndrome | NA | renal colic, abdomen pain | NA | | | | | | | |
| P77 | M | 56 | 21 | O | PKD | CRF | NA | NA | NA | | | | | | | |

| | | | | | | | | | | | | | | | | |
|---|---|---|---|---|---|---|---|---|---|---|---|---|---|---|---|---|
| P85 | M | 13 | 8 | O | Recurrent renal stone and pain | - | PKD on father and sibling | Vitamin D deficiency | NA | | | | | | | |
| P87 | M | 45 | 42 | O | Frequent urination, Pain when urinating | Tubulointerstitial Disease | NA | NA | NA | | | | | | | |
| P92 | F | 17 | 14 | O | Tubulointerstitial Disease | Renal Colic | - | - | NA | | | | | | | |
| P102 | M | 31 | 17 | O | Renal colic | Nephrolithiasis, cortical cyst | Brother and uncle(m) with CKD | fibromyalgia | yes | SLC3A1 | c.1973dup (p.Gln659ProfsTer7) | frameshift | HM | AD, AR | LP (PVS1, PM2) | Novel |
| P103 | F | 21 | 8 | O | Vomiting, renal colic | Nephrolithiasis, cystinuria | - | NA | third degree | SLC3A1 | c.647C>T (p.Thr216Met) | missense | HM | AD, AR | P (PS4, PS3, PM2, PM5, PM1, PP2, PP3) | rs369641941 |
| P104 | M | 41 | 41 | O | Incidental | chronic renal failure | CKD in older sister | - | yes | FAN1<br>LIPC | c.3004G>A (p.Glu1002Lys) | missense | HM | AR | VUS (PM2, PP3) | Novel |
| P105 | F | 14 | 9 | O | Abdominal pain, Joint pain | proteinuria | Nephrectomy history in her aunt | JIA | - | ACTN4 | c.1632G>A (p.Met544Ile) | missense | HT | AD | VUS (PM2, PP2) | Novel |
| P106 | M | 18 | 8 | O | CKD | CKD, Bilateral VUR | - | joint laxity | - | LRP5 | c.4268C>T (p.Pro1423Leu) | missense | HT | AD | VUS (PM2, PP3) | rs748143518 |
| P107 | M | 10 | 5 | O | proteinuria | proteinuria | - | - | - | OPLAH<br>MEFV<br>EK<br>BULGU:SPINK1 | c.3496_3498delCGGinsA (p.Gly1167LeufsTer21) | frameshift | HT | AD, AR | LP (PVS1, PM2) | Novel |
| P108 | M | 13 | 4 | O | edema | nephrotic syndrome | siblings with DM-1 | | - | PAX2<br>EK<br>BULGU:MEFV | c.673C>T (p.Arg225Trp) | missense | HT | AD | VUS (PM2, PP2, PP3) | rs142737641 |
| P109 | F | 14 | 5 | O | hematuria | bilateral nephrolithiasis | Nephrolithiasis in her father and brother | Hyperoxaluria | third degree | HOGA1 | c.569C>T (p.Pro190Leu) | missense | HM | AR | P (PS3, PM1, PM2, PM3, PM5, PP1,PP2, PP3) | rs202047589 |
| P111 | M | 4 | 1 | O | Detected in postnatal follow-ups | proteinuria, sclerosing glomerulopathy | - | - | fourth degree | CUBN | c.4689_4690delTAinsAT (p.Cys1563Ter) | Nonsense | HM | AR | P (PVS1, PM2, PM3) | rs2131697094 |
| P113 | F | 44 | 41 | O | proteinuria | proteinuria | proteinuria in her father, brother and sister. Renal transplantation in cousin | ischemic stroke | - | MEFV | c.2080A>G (p.Met694Val) | Missense | HT | AD, AR | P (PS1, PS4, PM1, PM2, PM5) | rs61752717 |
| P114 | F | 5 | 5m | O | development delay | BARTTER SYNDROME | Brother same phenotype | febrile convulsion | yes | CLCNKB | c.371C>T (p.Pro124Leu) | Missense | HM | AD, DR | LP (PM2, PP2, PP3) | Novel |

| | | | | | | | | | | | | | | | | |
|---|---|---|---|---|---|---|---|---|---|---|---|---|---|---|---|---|
| P116 | F | 1 | 0 | O | Multicystic dysplastic kidney | Multicystic dysplastic kidney | Multiple renal cyst in father | - | - | HNF1B ABCC8 | c.544+3_544+6del (p.?) | Splice loss | HT | AD | LP (PS4, PM2, PP1, PP3) | rs2033925649 |
| P118 | M | 13 | 2 | O | postnatal vomiting attacks | Distal renal tubular acidosis | - | Sensorineural hearing loss, GERD | yes | ATP6V1B1 | c.118+5G>T (p.?) | Nonsense | HM | AR | VUS (PM2, PP3) | Novel |
| P119 | F | | NA | O | Glucosuria, Hypouricemia | Glucosuria, Hypouricemia | Family history of diabetes mellitus | - | yes | SLC2A2 | c.589G>A (p.Val197Ile) | Missense | HM | AR, AD | VUS (PM2, PM5) | rs121909741 |
| P120 | M | 23 | 20 | O | Acute Renal Failure | HYPEROXALURIA | NA | NA | second degree | AGXT | c.584T>G (p.Met195Arg) | Missense | HM | AR | P (PS3, PM1, PM2, PM3, PM5, PP2, PP3) | rs180177244 |
| P121 | F | 16 | 14 | O | Nephrocalcinosis | Nephrocalcinosis | - | NA | - | CYP24A1 | c.428_430del c.364G>A (p.Glu143del) p.(Glu122Lys) | Frameshift Missense | Comp HT | AR | LP (PM2, PM4, PP5) VUS (PM2) | rs777676129 rs762334108 |
| P122 | F | 11 | 9 | O | Elevated creatinine | CKD | CKD in father and nephrolithiasis in some individuals | - | - | HNF1B | c.1406_1413del (p.Leu469ArgfsTer79) | Frameshift | HT | AD | LP (PVS1, PM2, PP5) | Novel |
| P123 | F | 28 | 31 | O | Dyspeptic Symptoms | Nephrocalcinosis | Nephrocalcinosıs ın grandfather | Elevated PTH | yes | KCNJ1 | c.601C>T (p.Leu201Phe) | Missense | HM | AR | LP (PM1, PM2, PM5, PP5) | rs200320892 |
| P124 | M | 15 | 10 | O | Nephrotic syndrome | Nephrotic syndrome, FSGS | NA | - | second degree | ALG8 | c.1179-2A>G (p.?) | splice site | HT | AD, AR | LP (PVS1, PM2) | Novel |
| P132 | M | 2 | 3m | O | Infantile nephronophthisis | Infantile nephronophthisis | Hıstory of an ex-sibling with a similar phenotype | Hyperoxaluria | second degree | AGXT | c.245G>A (p.Gly82Glu) | Missense | HM | AR | P (PM3, PS3, PM1, PP2, PM2, PM5, PP3) | rs121908522 |
| P133 | F | 41 | 33 | O | Glucosuria | Glucosuria | Family history of diabetes mellitus | - | second degree | SLC2A2 | c.589G>A (p.Val197Ile) | Missense | HM | AR | VUS (PM2, PM5) | rs121909741 |
| P134 | F | ex | newborn | O | PEROXISOME BIOGENESIS DISORDER | Abnormal RFT | - | Seizure, respiratuary failure, Elevated LFT | third degree | PEX12 | c.642del (p.Lys214AsnfsTer7) | Frameshift | HM | AR | LP (PVS1, PM2) | Novel |
| P135 | M | 36 | 30 | O | Incidental, URTI | CRF | Father in en stage renal failure (4. decade ex) and child in CKD | - | - | PAX2 | c.43+5G>A (p.?) | SPLİCE-SİTE | HT | AD | LP (PM2, PP3, PM5) | rs878853000 |
| P136 | F | 23 | 17 | O | FSGS | FSGS | Mother had CKD | - | - | INF2 | c.550G>A (p.Glu184Lys) | Missense | HT | AD | P (PP1, PS3, PS2, PM2, PP3) | rs1566778676 |
| P137 | M | 12 | 2 | O | generalized edema | Nephrotic syndrome 2/20 glomeruli are globally sclerotic. | Apert syndrome in a sibling | - | - | LMX1B | c.237G>T (p.Glu79Asp) | Missense | HT | AD | VUS (PM1,PM2,PP2) | Novel |
| P141 | F | 6 | 1 | O | Dyspnea | Acute renal failure (HUS) | - | Thrombocytopenia | - | CFH | c.908G>A (p.Arg303Gln) | Missense | HT | AD, AR | VUS (PM2) | rs76640858 |

| | | | | | | | | | | | | | | | | |
|---|---|---|---|---|---|---|---|---|---|---|---|---|---|---|---|---|
| P144 | M | 16 | 13 | O | Developmental Delay | CKD | CKD in two siblings | Developmental Delay | Second Degree | NPHP1 | DEL:chr2:110123535-110205223 (p.?) | CNV | HM | AR | P (1A, 2A-2E, 3A, 4L-4O) | Novel, RCV003327633.2 |

M: Male, F: Female, P: Patient, DC: Disease Classification, AS: Alport Syndrome, PKD: Polycystic Kidney Disease, CKD: Chronic Kidney Disease, HM: Homozygous, HT: Heterozygous, AD: Autosomal Dominant, AR: Autosomal Recessive, O: Other
